# Exosomes in Ocular Health: Recent Insights into Pathology, Diagnostic Applications and Therapeutic Functions

**DOI:** 10.3390/biomedicines13010233

**Published:** 2025-01-19

**Authors:** Noelia Blanco-Agudín, Suhui Ye, Sara González-Fernández, Ignacio Alcalde, Jesús Merayo-Lloves, Luis M. Quirós

**Affiliations:** 1 Department of Functional Biology, University of Oviedo, 33006 Oviedo, Spain; blancoanoelia@uniovi.es (N.B.-A.); yesuhui@uniovi.es (S.Y.); saragonzalezfernandez7@gmail.com (S.G.-F.); 2 Instituto Universitario Fernández-Vega, Fundación de Investigación Oftalmológica, University of Oviedo, 33012 Oviedo, Spain; nacho.alcalde@fio.as; 3 Instituto de Investigación Sanitaria del Principado de Asturias (ISPA), 33011 Oviedo, Spain

**Keywords:** exosome, extracellular vesicle, eye, ocular physiology, ocular pathology, ocular biomarker

## Abstract

Exosomes are extracellular vesicles ranging from 30 to 150 nm in diameter that contain proteins, nucleic acids and other molecules. Produced by virtually all cell types, they travel throughout the body until they reach their target, where they can trigger a wide variety of effects by transferring the molecular cargo to recipient cells. In the context of ocular physiology, exosomes play a very important role in embryological development, the regulation of homeostasis and the immune system, which is crucial for normal vision. Consequently, in pathological situations, exosomes also undergo modifications in terms of quantity, composition and content, depending on the etiology of the disease. However, the mechanisms by which exosomes contribute to ocular pathology has not yet been studied in depth, and many questions remain unanswered. This review aims to summarize the most recent knowledge on the function of exosomes in the ocular system in healthy individuals and the role they play during pathological processes of a degenerative, infectious, neurodegenerative, vascular and inflammatory nature, such as keratoconus, keratitis, glaucoma, diabetic retinopathy and uveitis. Furthermore, given their unique characteristics, their potential as diagnostic biomarkers or therapeutic agents and their application in clinical ophthalmology are also explored, along with the main limitations that researchers face today in the field.

## 1. Introduction

Extracellular vesicles (EVs) are membranous nanovesicles composed of a lipid bilayer produced and are released into the extracellular space by nearly all cell types, so they can appear in body fluids such as blood, saliva or tears [1]. They are classified according to their size and origin into three main groups: exosomes, with a diameter of 30 to 150 nm, which are of endocytic origin; microvesicles, ranging from 100 to 1000 nm in diameter, which are released directly from the plasma membrane; and finally apoptotic bodies, which are larger than 1 µm in diameter. The importance of exosomes lies not only in the content they carry inside but also in their ability to act as vehicles of long-distance communication between cells [2].

### 1.1. Brief History of the Discovery of Exosomes

The discovery of exosomes has its roots in blood coagulation research. The first appearance in the scientific literature of what would later be renamed exosomes dates back to 1946. Chargaff and West, two New Yorker researchers, observed that when blood was centrifuged at high centrifugal force, a fraction with coagulant properties was sedimented, which they attributed to small degradation products of red blood cells [3]. Years later, publications with electron microscopy images showing lipid membrane structures began to appear, which were called microparticles at that time, such as the research conducted by Sun in 1966 on alveolar cells [4] or by Crawford in 1971 on platelet-free plasma [5]. However, it was not until the 1980s that the term exosome was coined when Harding and Johnstone independently published two articles on reticulocyte maturation. Both observed the formation of vesicles released from the surface into the cell lumen, thanks to which the cells secreted waste cytoplasmic content [6,7]. Since then, knowledge and research in this field have grown exponentially, and exosomes have evolved from being considered forms of waste to vehicles of cellular communication, whose implications in human physiology and pathology are still largely to be discovered.

### 1.2. Biogenesis, Cargo and Molecular Components of Exosomes

The biogenesis of exosomes begins with the formation of early endosomes, which result from the invagination of the plasma membrane. These endosomes will subsequently mature into multivesicular bodies (MVBs), within which intraluminal vesicles are also generated by invagination. In this process, the ESCRT complex (Endosomal Sorting Complexes Required for Transport) is involved [8]. The ESCRT machinery consists of four complexes (ESCRT-0 to ESCRT-III) and a series of accessory proteins, which not only contribute to their biogenesis but also participate in their specific molecular cargo loading [9]. Subsequently, the MVBs are transported along the cytoskeleton to the plasma membrane, where they fuse and release intraluminal vesicles into the extracellular space as exosomes, a process mediated by proteins of the SNARE complex [2].

The composition of exosomes is highly diverse and depends on the physiological or pathological state of the cell. The outer membrane is lipid-based, mainly composed of phospholipids, cholesterol and ceramides. Intercalated within the lipid bilayer are membrane proteins such as tetraspanins CD9, CD63 and CD81, whose presence is shared by a large number of exosomes from different cellular origins. Other important molecules that can also be found in these EVs include growth factors, cytokines, and cell surface proteoglycans (PGs) [10], as well as nucleic acids, including DNA and different RNA species, such as miRNA or mRNA [8].

The loading of exosomal content takes place before the formation of endosomes and follows distinct pathways depending on the molecule. The protein loading mechanism is the best known and described to date. Generally, proteins must be monoubiquitinated in order to be recognized by the ESCRT complex, and they will subsequently be deubiquitinated and incorporated into the vesicle. In the case of RNA, there are various proteins called RNA-binding proteins, which facilitate its loading into exosomes [11].

Once exosomes have been loaded with their specific content carefully selected based on the physiological state of the cell of origin, they will be transported through the cytoplasm to the cell membrane. At this point, through a highly regulated process of exocytosis, the exosomes will fuse with the plasma membrane and are released into the extracellular space.

### 1.3. Mechanisms of Action and Biological Functions

Once released into the extracellular space, exosomes begin their journey through different body fluids and tissues until they reach their target. Some of the exosomes present in the medium are non-specifically captured by cells [8]. However, it has also been described that the composition of the exosome membrane and the extracellular matrix (ECM) of the target cell play a crucial role in determining its fate. This is particularly relevant in pathological contexts. For example, studies involving exosomes derived from cancer cells have revealed that these vesicles travel to specific organs where they promote the formation of pre-metastatic niches [12,13]. On the other hand, molecules such as PGs, especially heparan sulfate PGs (HSPGs), present both in the ECM and on the surface of exosomes, playing fundamental roles in specific binding and subsequent endocytosis [10,14]. Moreover, exosomes exhibit a certain prevalence for binding to cells of the same origin [15], thus participating in paracrine communication. Conversely, the presence of certain surface proteins can also act by significantly decreasing their binding to a specific cell type [16].

The primary and fundamental function of exosomes is intercellular communication and the transmission of information to both adjacent and distant cells. By releasing their content into the target cell, exosomes are capable of inducing physiological changes and participating in processes such as cellular homeostasis, tissue formation and repair, or immune system stimulation. However, as regulators of essential functions in the body, exosomes also play a crucial role in pathological situations. A well-analyzed example is cancer, where numerous studies have shown that the cargo of exosomes produced by tumor cells can interact with the immune system to prevent the recognition of these cells, contributing to immune evasion [17,18]. Another case is infectious pathologies, where exosomes can serve as a vehicle for the transmission of viral molecules, thus avoiding being recognized by the organism [19,20].

After understanding the mechanisms of action and biological functions of exosomes, it is essential to analyze their growing importance in biomedical research, where they are emerging as promising tools for the understanding of various diseases.

### 1.4. Importance of Exosomes in Biomedical Research

Their role in pathological conditions positions exosomes as nanoparticles with significant potential to be exploited in two of the basic pillars of medicine: diagnosis and treatment.

Exosomes are excellent candidates to act as biomarkers in different pathologies. Due to the fact that they are produced by nearly all cell types, they contain specific and characteristic cargo that will depend on the physiological or pathological state of the cell that produces it, they appear in many of the most commonly used bodily fluids in clinical practice and exosomes have been exploited both for the diagnosis and prognosis of diseases. Numerous studies have linked the presence of certain exosomal miRNAs with cardiovascular diseases [21], neurodegenerative disorders [22] and even the type and progression of cancer [23].

On the other hand, thanks to numerous advances in biotechnology, the use of exosomes for the treatment and prevention of diseases has been proposed, in what is known as cell-free therapy. Currently, drug delivery systems for ocular applications include carriers such as liposomes, nanoparticles or micelles. However, these carriers often face significant limitations such as low bioavailability, short retention time in ocular tissues and limited ability to penetrate deeper structures like the posterior segment of the eye. Additionally, some carriers may cause local irritation or trigger immune responses because of the low biocompatibility. In contrast, exosomes offer advantages due to their natural ability to penetrate biological barriers and capacity for targeted delivery. These EVs act as true “packages”, capable of traveling through the bloodstream in a stable form, without being phagocytosed or destroyed by the immune system, thus preventing their content from degradation and even being able to cross the blood–brain barrier [24]. Through bioengineering, the surface and content of exosomes can be altered, and they can be loaded with different therapeutic compounds, without the risk of immune rejection. One such example is their use in the development of new vaccines. Hence, during the SARS-CoV-2 pandemic of 2020, numerous investigations began to use viral antigens to modify exosomes and trigger the immune response [25].

However, these vesicles also present a series of limitations for therapeutic use. The first of these is the lack of a standardized exosome purification protocol common to all laboratories. In addition, low yields in their production and extraction, as well as challenges in the modification of their content, are some of the major drawbacks faced by researchers [24,26].

### 1.5. Justification of the Relevance of Studying Exosomes in Ocular Pathologies and Objectives of the Review

**Figure 1:**
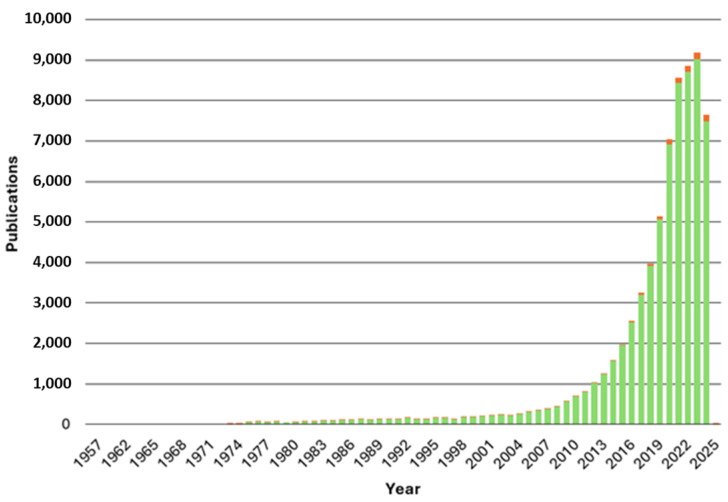
Number of publications in PubMed retrieved up to October 2024 with the keywords (*exosome*) OR (*extracellular vesicle*) in green. In red, publications retrieved with the keywords ((*exosome*) OR (*extracellular vesicle*)) AND ((*eye*) OR (*ocular*)).

The above data highlight the relevance that exosomes have been acquiring since their discovery in various fields of medicine. Most of the research carried out has focused on the role of these vesicles in commonly studied pathologies such as cancer and cardiovascular or neurodegenerative diseases. However, ocular pathology also has a great impact on society. In fact, the World Health Organization estimates that every person will suffer at least one ocular condition in their lifetime and that 2.2 billion people in the world have some visual impairment (https://www.who.int/news-room/fact-sheets/detail/blindness-and-visual-impairment, accessed on 3 October 2024). For this reason, research on exosomes in the field of ophthalmology has been progressively increasing in recent years (Figure 1), although it is still far from reaching the levels seen in cancer or cardiac pathology.

Therefore, the objective of this review arises from the need to gather the current knowledge on the role played by these vesicles in the normal physiology of the eye and during pathological processes of different natures. Finally, an overview will be provided of the current understanding of the use of exosomes in the diagnosis and treatment focused on ocular pathology.

## 2. Exosomes in Ocular System

The eye is the visual organ responsible for perceiving light. The eyeball is covered by a hard, white layer of tissue called the sclera, and its interior contains the vitreous humor. The outer surface of the eye is protected by a transparent membrane known as the conjunctiva. The cornea is an avascular dome-shaped protuberance that appears at the front of the eye, and it is separated from the conjunctiva by the corneal limbus, a reservoir of stem cells. It is responsible for directing light rays through the pupil to the retina thanks to the lens. The iris, the colored part of the eye, controls the size of the pupil and protects the retina from excess light. The anterior and posterior chambers of the eye are located between the cornea and the lens, and they are filled with aqueous humor, which maintains intraocular pressure and provides nutrients to the cornea and lens. In addition, it is also worth noting that the eye is permanently lubricated and protected thanks to the tear film [27].

**Figure 2:**
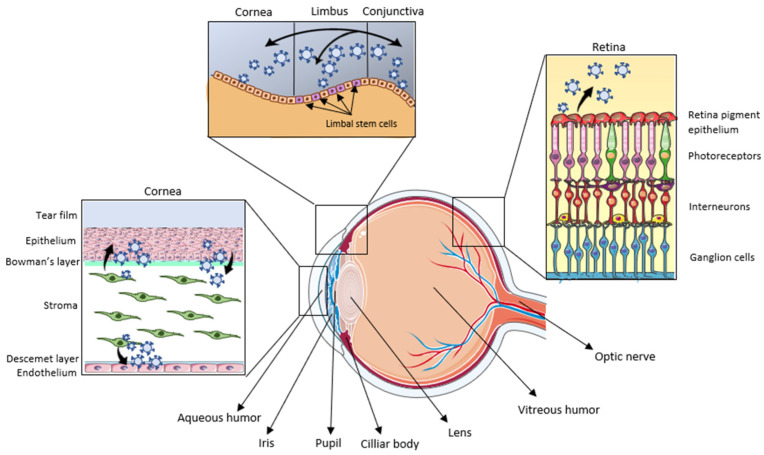
Schematic summary of the structure of the human eye, highlighting the tissues in which the presence of exosomes has been described in this review: the cornea, the limbus, the conjunctiva and the retina. In the cornea, there is an active exosomal communication between epithelium, stroma and endothelium. In addition, the cornea communicates with the conjunctiva through vesicles, which can also act on the limbus that separates them. In the retina, the production of exosomes has been described as being released by epithelial cells. Modified from https://smart.servier.com/, accessed on 7 November 2024.

The presence of exosomes has been described in most of the tissues that constitute the eye, such as the retina [28,29], conjunctiva [30], lens [31] or cornea [32,33,34], but also in ocular fluids such as the vitreous humor [35], aqueous humor [36] and tears [37] (Figure 2). The latter is also the most relevant and promising given its easy access, which makes it a potential non-invasive source of biomarkers.

One of the essential functions of exosomes in ocular physiology is communication between the different parts of the eye, with special relevance in the cornea and the retina. Thus, the exosomes secreted by corneal fibroblasts act on the endothelial cells of the cornea, inducing their proliferation and therefore cornea neovascularization [38], which is also a fundamental process in wound repair. Furthermore, there is a very close bidirectional cell–cell communication between the corneal epithelium and stroma. Exosomes secreted by epithelial cells, rich in thrombospondin-1, induce fibroblast differentiation into myofibroblasts, which has important implications for both the maintenance of homeostasis and tissue repair [33], by promoting ECM remodeling [39]. Likewise, the presence of laminins or HSPGs has also been detected in these exosomes [32], suggesting that they could be one of the ways in which cells release ECM components into the extracellular space in the eye, as has been already described in other tissues such as bone [40]. Conversely, the specific cargo of the exosomes produced by myofibroblasts is capable of inducing the motility, proliferation and migration of corneal epithelial cells [41]. Furthermore, exosomes secreted by corneal tissues can also act on adjacent tissues. For example, vesicles secreted by the corneal epithelium are able to travel through the tear film and act on the conjunctiva. The transdifferentiation between these two cell types has been described as a consequence of the bidirectional transfer of exosomes [30]. Interestingly, an enrichment of miR-9 has also been described in exosomes from conjunctival cells. This is of particular interest since this miRNA acts as an antagonist of HES-1, a repressor of corneal stem cell differentiation [42]. Therefore, the high levels of this miRNA in conjunctival exosomes could suggest that exosomes are able to induce the differentiation of corneal limbal cell precursors. These limbal stem cells also participate in the renovation of their own ECM by specifically loading exosomes with a large number of proteins related to its remodeling and cell–ECM communication [43].

On the other hand, the ability of exosomes to orchestrate the formation of the retina during embryological development has been described [44]. Although the vast majority of human cells can produce exosomes under physiological situations, it has not yet been demonstrated that healthy retinal neurons can generate them. Instead, numerous investigations in recent years have associated the production of EVs by retinal photoreceptors with tissue degeneration, potentially acting as a mechanism for expelling unwanted proteins produced as a consequence of cellular malfunction [28]. However, the ability of retinal epithelial cells to produce exosomes is widely recognized. In fact, it has been shown that these vesicles have protective properties against degeneration by inhibiting apoptosis and oxidative damage [45], which has great relevance in the field of therapies for degenerative retinal diseases.

Another key process in which exosomes participate, as a consequence of their ability to act as an intermediary in intercellular communication, is immunoregulation, widely described in relation to homeostasis maintenance in other tissues and various pathologies [17,18,46]. In the eye, the regulation of the immune system is particularly important given the immunological privilege it possesses, as exosomes intervene to maintain this immune homeostasis. In this regard, the ability of retinal epithelial cells to produce vesicles in vitro that reduce immune activity by acting on monocytes and changing their phenotype to a non-inflammatory state has been described [47]. In addition, the presence of αβ-crystallin in the exosomes secreted in vivo by these retinal epithelial cells has also been described, which is a negative regulator of both the innate and adaptive immune system [48,49].

## 3. Exosomes in Ocular Pathology

Since exosomes are a reflection of the state of the cell that produced them, they play a vital role not only in the regulation of ocular homeostasis but also in the onset and development of diseases of a different etiology. This makes them very interesting from a clinical perspective. The following sections address the importance of exosomes in different ocular pathologies and also focus on their nature. The exosomes are summarized in Table 1.

### 3.1. Keratoconus: A Degenerative Disease

Degenerative diseases are one of the hot spots of study in the field of exosomes. Traditionally, most research has focused on musculoskeletal degenerative pathologies. Thus, it has been described that these EVs can induce damage to cartilage, muscle and bone by transporting inflammatory molecules and various RNA species that act on cells producing different effects, such as proliferation, autophagy or apoptosis. The regulation of this microenvironment can even lead to the destruction of the ECM, a sign of degeneration [50]. Keratoconus is one of the most common degenerative ocular pathologies in the world, which can ultimately lead to blindness. It is characterized by a progressive decrease in the thickness of the cornea, which makes it acquire the typical conical shape [51]. Although it was traditionally considered a non-inflammatory disease, this dogma has been changing over the last decade, and numerous studies from different approaches claim that this pathology also alters inflammatory mediators [52]. At the histological level, the corneal stroma suffers the most significant transformations due to the degradation and modification of the keratocytes’ behavior. The internal structure of the stroma ECM is therefore affected in this pathology, primarily due to a disorder of collagen fibers and a decrease in HSPGs [53,54]. Furthermore, the epithelium appears altered as it loses its uniformity and undergoes degeneration [55].

Since exosomes participate in ECM remodeling in healthy individuals, analyzing these vesicles in patients with keratoconus can reveal interesting results. For example, alterations of the protein cargo have been described in patients when compared to exosomes from corneal stromal cells of healthy individuals, particularly affecting proteins related to adhesion and migration, such as ACTB or SERPINE1 [56]. In addition, these studies also revealed the overexpression of miR-184 in the exosomes of patients, which is significant since a mutation in this sequence has been linked to severe keratoconus and cataracts [57]. Vesicles generated by this cell type also exhibit a decrease in tetraspanins, such as CD9 or CD63 [56,58]. These molecules are associated with different cellular processes, such as cell adhesion or immune response in the case of CD9 or exosome synthesis and membrane transport in the case of CD63. This suggests that the production of exosomes by stromal cells is reduced in keratoconus [58]. On the other hand, apoptosis located in the corneal stroma also plays a fundamental role and has been related to the degeneration of the cornea in this pathology [51]. The presence of miRNAs that act by silencing apoptosis repressor genes has been described in exosomes produced by fibroblasts of patients with keratoconus, miRNAs that are absent in exosomes from healthy people [59]. At the histological level, these differences in their content result in the generation of different responses, depending on the origin and physiological or pathological state of the cell that produced them. Thus, exosomes from a diseased stroma induce a decrease in the proliferation and migration of stromal cells, an effect that is not observed when the stroma is exposed to healthy exosomes. However, on corneal epithelial cells, diseased vesicles are capable of producing an increase in their migration [56]. The importance of this lies in the fact that keratoconus not only produces changes at the stroma level but also the Bowman’s membrane that separates it from the epithelium is also compromised [60]. This would allow stromal exosomes to travel to the epithelial cells and induce different modifications, further contributing to the progression of the pathology.

### 3.2. Microbial Keratitis: An Infectious Disease

Infectious keratitis is defined as inflammation of the cornea due to its invasion by pathogenic microorganisms such as viruses, bacteria, fungi and amoebas. Depending on the causative agent, it can result in the total destruction of the corneal structures and blindness [61]. In infectious pathologies, exosomes can act as a double-edged sword [62]. On the one hand, exosomes produced by infected cells would function as carriers of pathogenic molecules such as bacterial toxins [63] or viral proteins [19,64]. As they are enveloped by an endogenous membrane, these molecules would be able to evade the individual’s immune system and subsequently contribute to the spread of the infection. On the other hand, exosomes can also develop anti-infectious functions by stimulating the immune response, enhancing antigen presentation and even inhibiting microbial proliferation and transmission [64,65,66].

Viral keratitis is currently the most studied form of the disease. One of the most common viruses causing keratitis is herpes simplex virus 1 (HSV-1), which can also remain in a latent state in neurons. When it infects retinal epithelial cells, this pathogen has been shown to modify the specific load of the exosomes produced by these cells by increasing the presence of neuroprotective and neurotrophic proteins within them. In this way, the growth of neuronal projections is enhanced, facilitating the spread of the infection to peripheral neurons [67]. In the context of herpetic keratitis, HSV-1 is also capable of altering exosomal biogenesis and cargo, producing an increased number of EVs and their enrichment in CD63, a tetraspanin involved in the production and trafficking of EVs [68,69]. In addition, HSV-1 uses the exosomes contained in the tear film as a reservoir to cause recurrent infections. Thus, it has been shown that these exosomes harbor viral proteins and even genes that are transferred between cells, resulting in recurrent infections [70]. As a result, current research is focused on the use of exosomes contained in the tear film as a very interesting non-invasive biomarker of pathology and even as a means for the application of antiviral drugs [69].

Among the bacteria that cause keratitis, *Pseudomonas aeruginosa* stands out. This Gram-negative pathogen can cause pathology in immunocompromised individuals and healthy people who present small wounds in the cornea or wear contact lenses [61]. Despite its frequency, limited information is available about the role played by human exosomes in the development of this bacterial infection. Unlike viral keratitis, no changes have been described in the quantity or size of the exosomes produced by infected cells, but rather in their content. The alterations of these vesicles could be aimed at modulating the innate immune response of the organism due to the presence of cytokines, such as IL-8, and other molecules mediating neutrophil activation, such as certain PGs. As in the case of HSV-1, these exosomes can also carry bacterial proteins that are crucial for virulence [71].

Despite being one of the most common ocular pathologies, little is still known about the role of exosomes in infectious processes affecting the eyeball. A complication that also arises in this case is the secretion of EVs by pathogenic bacteria, which are of similar size and density as exosomes and also participate in the modulation of keratitis [72]. Therefore, further research is still needed in this field to unravel not only the role played by EVs in keratitis but also the bidirectional regulation and communication established between these two kingdoms of life.

### 3.3. Glaucoma: A Neurodegenerative Disease

Neurodegenerative diseases are one of the fields in which exosomes are most studied. Under these pathological conditions, it has been shown that exosomes serve as a communication mechanism between neuronal cells. The existence of an aberrant synthesis of EVs has also been described, transporting misfolded proteins that further spread the disease [73,74]. However, most of the research on neurodegenerative diseases has focused on Alzheimer’s and Parkinson’s diseases. Yet, little is known about the role that these vesicles play in ocular neurodegenerative pathologies, such as glaucoma. The term glaucoma encompasses a group of diseases that produce a progressive loss of retinal ganglion cells and damage to the optic nerve, which leads to vision loss. Among the risk factors that can trigger this pathology is the increase in intraocular pressure due to impaired drainage of the aqueous humor. Under physiological conditions, this fluid is produced mainly by the non-pigmented ciliary epithelium of the ciliary body, flows into the posterior chamber of the eye and is finally drained through a porous structure called the trabecular meshwork [75].

The composition of the trabecular meshwork ECM is key to its correct functioning. An increase in the deposit of molecules in the ECM has been reported in glaucoma patients, which alters its dynamics and disrupts the normal flow of the aqueous humor [76,77]. Since exosomes secreted by trabecular meshwork cells participate in the normal remodeling of the ECM, alterations in its content may contribute to this pathology. First, the comparison of aqueous humor between glaucoma patients and healthy individuals revealed an increase in the presence of exosomes in the patients [78], being significantly smaller [79], in addition to having a greater presence of the three main tetraspanins. This increase is especially relevant in the case of CD63, which was found to be 50% more abundant in the glaucoma samples compared to controls [78]. It is interesting that this smaller size in glaucoma patients can influence their cargo capacity and lead to greater degradation of the collagen present in the trabecular meshwork [79]. At the proteomic level, the comparison between both types of EVs also shows a large number of differences. Proof of this is the study carried out by McDonnell and colleagues on exosomes from the trabecular meshwork, in which they only found 35 proteins in common among the 100 most abundant of both vesicles. Notably, fibronectin and EDIL3 were significantly reduced in vesicles from meshwork cells of glaucoma patients compared to healthy controls. These proteins are particularly relevant since they are ECM components and also participate in cell–ECM migration and adhesion processes. Interestingly, these researchers also detected a higher presence of fibronectin in cell lysates from glaucoma patients compared to healthy controls, hypothesizing that this lack of fibronectin elimination by exosomes may lead to its accumulation in the trabecular meshwork, increasing the resistance to aqueous humor flow [80].

**Figure 3:**
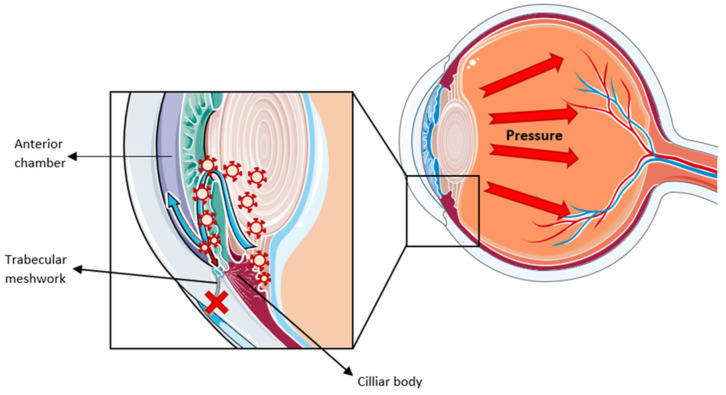
Diagram illustrating intraocular pressure in the anterior chamber of the eye. Aqueous humor flows from the ciliary body into the anterior chamber and out through the trabecular meshwork. In glaucoma, exosomes are able to induce pathological changes, such as dysfunction of the trabecular meshwork, which can lead to increased intraocular pressure, a hallmark of glaucoma progression. Modified from https://smart.servier.com/, accessed on 7 November 2024.

On the other hand, this pathology is another clear example of how exosomes act as a communication mechanism between different cell types, in this case between cells of the non-pigmented ciliary epithelium and those of the ciliary body of the trabecular meshwork [81]. Exosomes produced by epithelial cells carry bioactive molecules involved in key processes in glaucoma, such as cell adhesion or ECM deposition. Thus, proteins such as PP2A phosphatase capable of acting on the canonical Wnt signaling pathway and inducing a decrease in the accumulation of β-catenin were detected [82]. This pathway holds special relevance in the context of the pathology since alterations in it have been related both to poor aqueous humor drainage and the development of glaucoma [83]. Another molecule of interest, identified by Lerner and colleagues, is miR-29, another negative regulator of the Wnt/β-catenin pathway [82]. Furthermore, histological analysis shows that the treatment of trabecular meshwork cells with exosomes derived from non-pigmented ciliary epithelial cells resulted in a decrease in the synthesis of type I collagen [84] and COL3A1 [82]. Since both the trabecular meshwork and the ciliary body are key structures in the regulation of aqueous humor flow, the existence of this communication between both through exosomes is key in pathological processes that affect this drainage system, such as glaucoma. Thus, the increased presence of exosomes in the aqueous humor could lead to greater degradation of the ECM of the trabecular meshwork, further enhancing the disease (Figure 3).

### 3.4. Diabetic Retinopathy: A Vascular Disease

Another prominent area of exosome research is vascular disease. Accordingly, it has been reported that the content of EVs produced by cardiomyocytes under hypoxic conditions, which would simulate ischemic heart disease, is rich in molecules that promote angiogenesis, inflammatory response and fibrosis, acting as mediators of reperfusion disease [85]. However, the ability of these exosomes to actively participate in tissue recovery after heart failure has also been described [86]. Diabetic retinopathy is a complication that affects almost one-third of patients with diabetes mellitus and is a leading cause of vision loss that can result in blindness [87]. Chronic high blood glucose levels damage the blood vessels that supply the retina, leading to capillary closure, hemorrhages, oxidative stress, hypoxia and ischemia in the initial phase, followed by abnormal and dysfunctional neovascularization of the retina and reperfusion injuries [88,89]. In this case, since diabetic retinopathy is considered a vascular condition and exosomes can travel long distances through body fluids to act on target tissues, there are numerous investigations that have focused on EVs detected not only locally in the eye but also systemically in the blood or other tissues.

It is noteworthy that the content of exosomes detected in plasma from patients with diabetic retinopathy is altered not only compared to healthy individuals but also compared to diabetic patients without this ocular pathology [90]. This gives an idea of the exosomal cargo specificity and to what extent they are able to reflect the cellular state. EVs produced from pathological cells also have the ability to act on pericytes and endothelial cells, inducing migration, increased vascular permeability and angiogenesis—characteristic processes of diabetic retinopathy that lead to the appearance of microvascular damage in the retina and the breakdown of the blood–retinal barrier [90,91]. Mazzeo and colleagues especially attributed these changes to three miRNAs: miR150-5p, miR21-3p and miR30b-5p, which have been previously related to characteristic manifestations of this disease [92,93,94]. Another study, in the same way, indicates that exosomes purified from the serum of patients with diabetic retinopathy produce an overexpression of the α subunit of the fibrinogen coagulation factor (FIBA) in endothelial cells [95], a key molecule in endothelial dysfunction in diabetic retinopathy [96]. In addition, EVs produced in this context also carry proinflammatory cytokines and molecules capable of activating the complement pathway, further contributing to creating a pro-pathological scenario [91,97].

At the local level, it has been demonstrated that exosomes secreted by retinal epithelial cells exposed to oxidative stress, a hallmark of diabetic retinopathy [89], generate a greater quantity of exosomes and promote the formation of new blood vessels by acting on endothelial cells. These exosomes carry higher amounts of the VEGF receptor, bound to their membranes and the mRNA encoding it inside them [98]. Interestingly, not only mRNAs and miRNAs are capable of actively participating in the regulation of physiological and pathological processes in humans, but also long non-coding RNAs (lncRNA), whose presence has already been reported in EVs, act as regulators of gene transcription, being able to influence the development of diseases [99]. In the case of diabetic retinopathy, there are several investigations that reveal alterations of lncRNAs [100], in which exosomes can act as messengers between cells. In this context, it has been seen that the exosomes present in the vitreous humor of diabetic patients with retinopathy are highly enriched in the lncRNA LOC100132249. When this lncRNA acts on the retinal endothelial cells, it is capable of inducing their proliferation and migration, leading again to neovascularization [101].

### 3.5. Uveitis: An Immune and Inflammatory Disease

Uveitis is defined as a group of intraocular diseases of different etiologies, which are often unknown but share their inflammatory nature. They can affect the iris, ciliary body and choroid [102], although they can also involve other structures such as the retina or optic nerve. In developed countries, the most common form of uveitis is autoimmune, which can appear alone or as a secondary condition to the onset of a systemic autoimmune disease [103]. Therefore, the importance of uveitis is due not only to its potential to cause total vision loss but also to the fact that it can be one of the first symptoms of other underlying pathologies, making timely and accurate diagnosis critically important. Since exosomes actively participate in regulating the immune system, the analysis of their alterations is of great interest when addressing the study of this and other autoinflammatory diseases. In this context, EVs secreted by or targeting T cells are of particular interest. For example, exosomes produced by tumor cells can act on T cells and inhibit their activation through PD-L1, an immune system suppressor, thus enhancing tumor growth [104].

In the case of uveitis, it has been shown that exosomes traveling in blood plasma carry inflammatory-regulating molecules, such as FSTL1 or TRIM21, capable of stimulating the production of proinflammatory cytokines or acting on T cells [105]. In addition to proteins, exosomes also contain miRNAs that block T cell proliferation, such as miR-410-3p, by targeting the cytokine CXCL5 [106]. Exosomes present in aqueous humor also harbor immune system-related proteins. Specifically, EVs derived from uveitis patients are enriched with complement cascade activating components, such as C1QB [107].

Histologically, it has been demonstrated that both T cells and monocytes from uveitis patients are regulated by exosomes secreted by the retinal pigment epithelium. Under inflammatory conditions, these EVs are capable of, on the one hand, inhibiting T cell proliferation and, on the other, enhancing the production of proinflammatory cytokines and even monocyte death [47]. Despite the established importance of exosomes in inflammatory pathology, little is still known about the role they play in the development of uveitis, and existing research focuses on their potential therapeutic applications. Many of these investigations concentrate on exploiting exosomes naturally or artificially loaded with interleukins as a mechanism to act on T cells, thereby attempting to reduce the development of uveitis [108,109]. For example, in murine models, Kang and colleagues demonstrated that exosomes produced by B cells, rich in IL-27, have the ability to decrease induced uveitis in mice by acting again on T cells [110].

**Table 1:** Summary of exosome alterations and their impact on human physiology in the pathologies discussed in this review.

Disease	Etiology	Cell Type Producing/ Fluid Studied	Alterations in EVs	Effect on Human Physiology	References
Keratoconus	Degenerative	Corneal stromal cells	Decreased detection Decreased CD9 and CD63 Apoptosis-, adhesion- and migration-related proteins and miRNAs	Decreased proliferation and migration of stromal cells Increased migration of epithelial cells	[56,57,58,59]
Microbial keratitis	Infectious	Tears	Viral: Increased detection Increased CD63 Viral proteins and genes	Viral: Reservoir Viral dissemination	[67,68,69,70,71]
		Corneal epithelial cells	Bacterial: Cytokines, bacterial proteins	Bacterial: Exacerbated innate immune system response	
Glaucoma	Neurodegenerative	Aqueous humor	Increased detection Increased CD63, CD9 and CD81 Smaller size		[78,79,80,82,84]
		Trabecular meshwork cells	Proteins and miRNAs targeting the Wnt/β-catenin pathway Decreased ECM proteins	Decreased ECM collagen synthesis	
Diabetic retinopathy	Vascular	Blood	miRNAs targeting angiogenesis processes, FIBA Cytokines	Increased cell permeability and angiogenesis Damage to the blood–retinal barrier	[90,91,95,98,100]
		Retinal pigment epithelial cells	Increased detection VEGFR		
		Vitreous humor	lncRNA	Endothelial cell proliferation and migration	
Uveitis	Immune	Blood plasma	Proinflammatory molecules and miRNAs targeting T cells	Inhibition of T cell proliferation	[47,105,106,107]
		Aqueous humor	Complement-activating molecules		

## 4. Diagnostic Potential of Exosomes in Ocular Pathologies

As discussed throughout this review, the unique characteristics of exosomes make them promising candidates to act as biomarkers of different ocular pathologies. Among these characteristics, the following stand out:

(a) The quantity and specific cargo contained in exosomes depend on the physiological or pathological state of the producing cell and therefore can be associated with a specific pathological condition [20].
(b) Its vesicle-shaped structure provides protection to its cargo against degradative enzymes that may be present in the extracellular medium [8].
(c) Exosomes are released to the outside and can be found in easily accessible body fluids, such as blood or tears, making them excellent non-invasive biomarkers [20,111].

Currently, strategies for diagnosing ocular diseases involve a range of tests to examine the eye, such as optical coherence tomography, color fundus photography and electroretinography. However, the main issue with these approaches is that they focus on structural alterations, require expensive and time-consuming equipment, and include a subjective component, as they rely on the expertise of the individual interpreting the results [112]. In contrast, as previously described, exosomes hold the potential to uncover the underlying mechanisms of diseases and facilitate personalized diagnostic approaches.

However, exosomes also present a series of limitations for their use in the diagnosis and prognosis of ocular pathology. At a theoretical level, the main problem is the lack of knowledge regarding the role they play not only in the disease but also in the healthy eye. In addition and as a result, there is no general consensus on which specific molecules can act as biomarkers in each pathology. At a practical level, the reality is that the isolation and purification rates of exosomes from ocular fluids are relatively low, and there are no standardized methods common to all laboratories that allow for obtaining high and reproducible yields [112]. For example, the concentration of exosomes in tears is typically around 10^8^ to 10⁹ particles/mL, which is relatively poor when compared to the concentration in blood serum, which can reach approximately 10¹³ particles/mL [20]. Moreover, the techniques for isolation, purification and characterization of exosomes such as ultracentrifugation, immunoaffinity capture and size-exclusion chromatography are highly heterogeneous today and vary greatly between laboratories. To address these and other issues, the International Society for Extracellular Vesicles (ISEV) established the first guidelines in 2014, integrating recommendations, considerations and protocols when working with EVs. In 2023, they presented its latest update to date, “Minimal information for studies of extracellular vesicles (MISEV2023): From basic to advanced approaches” [113]. Efforts should prioritize standardizing isolation and characterization protocols to ensure reproducibility and comparability across studies. In addition, more advanced technologies like high-resolution imaging, single-particle tracking and comprehensive proteomic and RNA profiling could enable a more thorough understanding of the use of exosomes as reliable diagnostic biomarkers [112].

Not only is the specific cargo of exosomes important in clinical applications, but the mere presence or absence of EVs, as well as the concentration at which they are detected, may also be subject to study, a characteristic that is perhaps more underestimated and less exploited. Accordingly, the production of EVs by the photoreceptors of the retina can indicate the presence of problems in the tissue, associated with the early stages of retinal degeneration [28]. However, the main practical limitation of this circumstance is that these vesicles are released into the vitreous humor, a fluid that is difficult to access and from which large volumes cannot be extracted, in addition to the lack of characterization of the exosomes present in it [44]. On the other hand, there are numerous investigations that have reported changes in the concentration of exosomes detected in patients with some ocular [58,68,69,90] and non-ocular [23,114] conditions. Therefore, this indicator represents a promising mechanism for clinical study in ophthalmology, although further studies still need to be developed and applied to research on other ocular pathologies.

However, most studies focus on identifying qualitative differences in the content of exosomes. In diseases affecting the anterior segment of the eye, tears will play a fundamental role in the diagnosis, since many of the EVs are released into the tear film, and it constitutes an easily accessible and non-invasive biofluid, which also does not usually present large amounts of proteins or other potentially contaminating waste substances. Consequently, exosomes contained in the tear film have been studied in numerous investigations from a multi-omic perspective to identify biomarkers of diverse pathologies, such as keratoconus [58], glaucoma [115], herpetic keratitis [69] or dry eye [116]. Although the normal volume of human tears ranges from 3 to 10 µL [117], the truth is that these EVs express higher levels of CD9 and CD63, two of the traditional exosome markers, compared to those found in plasma [118], which would facilitate their detection and monitoring in diagnostic methods. In addition, in recent years, innovative and highly ingenious techniques have emerged to overcome this challenge. One such example is iTears, a rapid isolation system for exosomes contained in tears and specially designed to work with small volumes based on the application of alternating negative pressure combined with immunodetection for subsequent analysis. Using this system, researchers were able to achieve concentrations 10 to 100 times higher, and they managed to describe the presence of a range of miRNAs and proteins that were altered in dry eye disease and diabetic retinopathy, although this system could potentially be applied to the identification of other ocular or systemic pathologies [119]. Another fluid of particular interest in the context of anterior segment pathology of the eye, particularly in glaucoma, is aqueous humor, although it is also gaining relevance in retinal diseases given the proximity of the tissue [75].

On the contrary, in diseases affecting the posterior segment of the eye, the biofluids that acquire the greatest relevance are vitreous humor and blood [112]. Since miRNAs are the most studied and widely used molecules as exosomal biomarkers today [24], numerous investigations focus on the detection of these molecules, their changes in EVs and their association with the specific pathology [90,106,120]. It is also worth highlighting the existence of clinical trials currently based on the application of exosomes as diagnostic methods for ophthalmological diseases. For example, the National Eye Institute is conducting a study entitled “The Vitreous Proteome and Inflammatory Mediators in Ocular Inflammatory Disease”, whose aim is to determine the cytokine content of exosomes present in the vitreous humor of patients with ocular inflammatory pathologies (NCT00331331). Additionally, blood serum or plasma has also been also the subject of clinical trials, with the objective of finding differences in miRNA or protein levels in exosomes from patients with diabetic retinopathy (NCT03264976, NCT06188013).

## 5. Therapeutic Potential of Exosomes in Ocular Pathologies

In addition to their potential application as biomarkers, the study of exosomes has been addressed for their use in the treatment of ocular pathologies. Beyond the characteristics mentioned above, other properties that contribute to their use in this field are as follows:

(a) Since exosomes are generated by their body cells, they have low toxicity and immunogenicity, even lower than the cells from which they come [112], making them unrecognized as foreign entities and preventing immunological rejection [26].
(b) Exosomes have the ability to penetrate biological barriers in the eye such as the blood–retinal barrier, which gives them an enormous advantage over other drugs [121].
(c) Their manipulation through bioengineering is relatively simple, and it can be performed on EVs obtained from the body fluids of the patient who will subsequently undergo treatment, ensuring high biocompatibility [26,74].

Like their application in diagnostics, exosomes also have a series of limitations for their use as therapeutic agents, in addition to those mentioned earlier. The first factor to take into account when conducting a study is the safety and potential side effects of using exosomes in the eye, mainly due to excessive intraocular proliferation and even interference with neural communication [122]. Furthermore, the route of administration and dosage also has a great influence, both on the distribution and on the rate at which exosomes are eliminated from the systemic circulation. Essentially, the application of these EVs for the treatment of ocular diseases is performed locally, either topically (the most commonly used method) or through subconjunctival, intravitreal or subretinal injection [112]. Finally, there are different strategies to address exosome cargo, their durability in circulation and surface modification so that they can target specific tissues more effectively [26]; each strategy has different success rates, advantages and disadvantages.

One of the most promising areas of application of exosomes is corneal regeneration, a multifactorial process involving proliferation, migration, angiogenesis or ECM remodeling. It has already been described that exosomes secreted by corneal cells have the capacity to induce neovascularization of the tissue following injury, epithelial regeneration and ECM modification [38,39,41]. Of particular importance in this context are exosomes isolated from mesenchymal stem cells (MSCs). Currently, since it is demonstrated that much of the therapeutic potential of these cells is actually exerted by the paracrine factors they secrete [123], there are many investigations based on the use of these EVs for the treatment of ocular diseases of different etiologies, and they are even the subject of various clinical trials [112,121]. In addition, they offer many advantages over the use of MSCs themselves in ocular therapy since these cells present a series of risks such as non-specific differentiation, immune rejection, intraocular hemorrhages and even vision loss [124], risks that exosomes lack. In this sense, exosomes isolated from MSCs from the cornea of cadaveric donors produced an increase in healing of more than 50% [125]. This effect is also shared by exosomes from MSCs from other niches [126,127]. The mechanism through which these exosomes act appears to be related to the regulation of the PTEN/PI3K/Akt pathway and the reduction in the synthesis of proinflammatory cytokines and apoptosis [127,128].

Retinal diseases are another major focus for exosome-based therapies. This tissue lacks the ability to regenerate on its own, which in pathological situations leads to irreversible vision loss [44]. For this reason, one of the main therapeutic approaches is cell transplantation to the eye. However, the use of exosomes presents advantages over the application of the cells themselves, and it also causes less damage to the structure of the organ during the operation. An example of this is the research carried out by Wang and colleagues in murine models of retinal degeneration. By applying exosomes purified from retinal epithelial cells, these researchers managed to reduce the presence of proinflammatory cytokines, oxidative damage and apoptosis, in addition to recording an improvement in the retinal cones, which resulted in a boost in visual capacity [45].

There are various strategies for modifying exosomes, which are not limited to loading them with therapeutic compounds but range from membrane modification to gene loading. First, the alteration of the content of the exosomes makes them more suitable and enhances the success of the therapy. Depending on the molecule loaded into the exosomes, a wide range of therapeutic applications becomes possible. For example, the use of neuroprotective peptides such as PACAP38 in exosomes derived from retinal ganglion cells led to a regeneration of the affected axons in rats with optic nerve damage and an improvement in the survival rate of the exosome-producing cells [129]. The modification of these EVs with KV11, an antiangiogenic peptide, resulted in a decrease in pathological neovascularization and blood leakage from the eye vessels in murine models of retinopathy. Interestingly, these researchers also compared the efficacy of transporting free KV11 versus in exosomes, and discovered that both the stability and its therapeutic effect were greater when the peptide was encapsulated within the vesicle [130]. Another possibility would be to treat exosomes with compounds that enhance their effects. One example is oridonin, a diterpenoid with anti-inflammatory properties that has been shown to protect the corneal epithelium from hyperosmolarity and its consequences in pathologies such as dry eye disease [131]. Additionally, it has been demonstrated that pretreating exosomes with oridonin enhances their positive effects on ischemia–reperfusion injury, making its use a promising therapeutic strategy to mitigate cellular damage induced by oxidative stress and inflammation [132]. On the other hand, another interesting strategy aimed at improving the affinity of EVs for their target involves modifying the surface membrane. Thus, the cyclic RGD peptide has a high affinity for retinal epithelial cells, so modifying the exosomal membrane with this molecule would enable more specific binding to this cell type in the treatment of retinal degeneration [112]. Regarding genetic modification, this can be carried out by regulating the state of the producing cell through transfection using plasmids or viral vectors, or by electroporation, with the aim of loading them with different species of RNA (miRNA, mRNA or siRNA). However, the main limitation of this technique is the difficulty of controlling the expression of the target gene, making it less specific [74,112]. Further studies are required to elucidate the exact molecular mechanisms involved and to optimize treatment protocols and the administration of exosomes in clinical models. Finally, an innovative and distinct strategy would involve not only modifying the exosomes themselves but also creating scaffolds that enable the proper diffusion of these EVs. An interesting example is the study conducted by Sun and collaborators for corneal epithelium healing. In their research, they not only modified exosomes to enrich them with miRNA 24-3p, a miRNA involved in cell migration, but also developed a thermosensitive hyaluronic acid hydrogel specifically designed for the controlled release of exosomes, particularly tailored for corneal regeneration in alkali burn models [133]. In this regard, a novel and promising approach to explore would be the development of new scaffolds specifically designed to optimize the precise delivery of exosomes in various therapeutic contexts.

Within retinal diseases, age-related macular degeneration (AMD) is one of the pathologies where the application of exosomes is rapidly gaining attention. This is primarily due to the fact that these vesicles play a dual role in the development of this condition. On the one hand, it has been demonstrated that exosomes secreted by the retinal pigment epithelium are enriched with components associated with the onset of AMD [134]. This leads to the formation of drusen, deposits that interfere with the exchange between the retinal epithelium and the choroid, resulting in oxidative stress. Notably, molecules such as annexins and CD63, characteristic components of exosomes, have been identified within drusen [29].

Consequently, experimental models have shown that exosomes derived from retinal cells can either promote or mitigate the damage associated with AMD, depending on their composition. One of the main challenges faced by researchers when addressing conditions in the posterior segment of the eye is achieving effective penetration to ensure that therapies reach their specific target with maximum efficiency [135]. Since oxidative stress is one of the most significant factors in this pathology, numerous studies focus on targeting the signaling pathways involved. For instance, it has been shown that exosomes produced by mesenchymal stem cells are capable of activating the Nrf2/Keap1 pathway, thereby protecting the RPE from oxidative damage by regulating homeostasis and innate immunity, and maintaining the stability of retinal structure [136].

Specific clinical trials are essential to validate the therapeutic potential of exosome-based therapies, focusing on targeting the hallmarks of the disease. For example, reducing retinal ganglion cell loss or intraocular pressure could be an effective approach to assess the efficacy of exosome-derived treatment in glaucoma. Similarly, for corneal diseases such as keratoconus or microbial keratitis, clinical studies could explore the role of exosomes in promoting wound healing and restoring corneal transparency.

## 6. Conclusions

Currently, EVs, particularly exosomes, have gained increasing importance in medicine. Initially considered mere waste substances, the scientific community has been able to overcome this early conception and focus on exploiting their full potential. Due to their ability to act as vehicles for cellular communication, their functions are key in a wide range of essential processes in the body, many of which are still being discovered. These processes include cellular maintenance, tissue remodeling or the regulation of the individual’s immune system. Particularly, and perhaps somewhat overlooked, exosomes play a fundamental role in regulating the normal physiology of the eye and maintaining its privileged immune status, being present in different ocular tissues and fluids, such as the cornea, retina, conjunctiva, vitreous humor, aqueous humor and tears. Therefore, in pathological conditions, exosomes undergo a series of alterations in the quantity, composition and content, reflecting the breakdown of ocular homeostasis. On many occasions, these alterations will depend on the nature of the pathology and are shared by diseases of the same etiology that affect other organs or tissues. Given their importance in ophthalmology, in recent years, their application to the diagnosis and therapy of ocular pathologies is increasing. The characteristics of exosomes make them excellent candidates to act as biomarkers of ocular pathology, as they can be detected in easily accessible biofluids. On the other hand, therapeutic application is also being widely studied for treating ocular disease, with particular interest in exosomes derived from MSCs, and is already translating into the more frequent appearance of clinical trials based on these EVs. Nonetheless, current exosome research still faces limitations, including the heterogeneity of isolation methods, challenges with reproducibility concerns, variations in experimental protocols and different technical approaches, which often result in discrepancies between studies, making it difficult to establish standardized methods. Given the limitations that have yet to be overcome, more studies need to be performed. Further research and knowledge, from a multidisciplinary perspective, are needed on the basic biology and translational medicine of exosomes.

## Data Availability

The data presented in this study are contained within the article.
